# What Forms, Maintains, and Changes the Boldness of Swimming Crabs (*Portunus trituberculatus*)?

**DOI:** 10.3390/ani12131618

**Published:** 2022-06-23

**Authors:** Boshan Zhu, Xianpeng Su, Weiping Yu, Fang Wang

**Affiliations:** 1 The Key Laboratory of Mariculture, Ministry of Education, Ocean University of China, 5 Yushan Road, Qingdao 266003, China; zhuboshan@stu.ouc.edu.cn (B.Z.); suxianpeng0405@163.com (X.S.); 2 School of Biological and Ecological Engineering, Dongying Vocational Institute, 129 Fuqian Street, Dongying 257091, China; adongzi@126.com

**Keywords:** *Portunus trituberculatus*, boldness, stability of personality, behavioral flexibility, energy metabolism, 5-HT

## Abstract

**Simple Summary:**

The formation and maintenance mechanism of personality is a hot topic in personality research. The boldness of the swimming crab (*Portunus trituberculatus*) has the greatest influence on its behavior, but the physiological factors leading to the stability of and difference in boldness are not yet clear. In this study, the factors affecting the formation and maintenance of and change in the boldness of swimming crabs are investigated from the perspectives of behavior, physiology, and neurotransmitters. The results show that the differences in boldness among crabs remain consistent for a long time and are closely related to the body’s energy and neurotransmitter metabolism.

**Abstract:**

Boldness of personality is an important theme in animal behavior and has significant ecological and evolutionary consequences. Studies on boldness in crustaceans typically focus on their behavior, while relatively few studies have focused on the formation and maintenance of and change in boldness, such as energy metabolism and neurotransmission. In this study, we measured the boldness of swimming crabs (*Portunus trituberculatus*) and analyzed the relationship between boldness and oxygen consumption rate, energy concentration, and the relative expression of energy-metabolism-related and 5-HT genes in mRNA. The results showed that boldness remained stable across repeated tests but changed under dangerous conditions. Swimming crabs could be divided into bold and shy individuals. Bold individuals consumed oxygen at a significantly higher rate than shy individuals. Lactate and glucose concentrations in hemolymph were significantly lower in bold individuals than in shy individuals, and mRNA relative expression of Na^+^/K^+^-ATPase and 5-HT genes was significantly higher in bold than in shy individuals. Preliminary results indicate that energy metabolism and neurotransmitters may underlie the formation and maintenance of personality characteristics of swimming crabs. Swimming crabs also exhibit behavioral flexibility in order to cope with risks. This may be an adaptation to their complex environments.

## 1. Introduction

Animal personality is the behavioral tendencies differing across individuals but consistent over time and across contexts within individuals [1]. Boldness is an evaluation of an animal’s willingness to take risks in order to obtain more food resources or reproductive opportunities and an aspect of personality that describes animals’ responses to potential risks in their habitat [2,3,4]. The fiddler crab (*Uca mjoebergi*) and the hermit crab (*Pagurus bernhardus*) are typical species in the study of boldness and have been studied widely [5,6]. In personality research, the stability of behavioral character is the consistency of behavioral differences among individuals over time and under different conditions [7]. Personality (stability in behavior) has been studied primarily in aquatic species such as crayfish (*Astacus astacus*) and fiddler crab (*U. mjoebergi*) [5,8]. Behavioral plasticity, in contrast, means that animals adapt to environmental changes (including adverse environments) by changing their behavior [9]. The complementary relationship between behavioral plasticity and personality stability is of great evolutionary significance [10]. With feedback mechanisms and survival strategies underpinned by particular trade-offs, some of the best contenders within a population have different personalities in the same environment.

A difference in energy metabolism is one internal basis for differences in animal personality [9]. Different metabolic rates represent differences in the concentrations of energy sources such as glycogen and glucose, which are required for physiological functions and behaviors. Generally, individuals with high metabolic rates move faster, explore more, and are more likely to be identified as bold individuals. This pattern has been observed in terrestrial animals such as great tits (*Parus major*) and lizards (*Zootoca vivipara*) [2,11] and aquatic animals such as Atlantic salmon (*Salmo salar*) and Arctic char (*Salvelimus alpinus*) [12]. Kucharski et al. and Velando et al. found that differences in animal personality are regulated by genes related to energy metabolism [13,14]. However, the relationship between the personalities of crustaceans and the regulation of energy metabolism has only been discussed in crayfish (*Procambarus clarkii*) and fiddler crab (*U. mjoebergi*) [15,16], and the relevant research needs to be expanded.

Swimming crab (*Portunus trituberculatus*), well known for aggression and boldness [4], is a typical species in the study of aquatic animal personality [4,17]. However, the physiological metabolism affecting boldness is still unclear. In order to answer this question—“What forms, maintains, and changes the boldness of swimming crab?”, we studied the boldness of swimming crab in captivity. Video recordings of three boldness measurements were used to quantify the boldness of swimming crabs, and the stability of boldness and behavioral plasticity was analyzed. In addition, to measure the stability of boldness when the environment changes, a simulated dangerous situation with hemolymph added was used in repeated measures. Energy-metabolism-related indicators, including oxygen consumption rates, concentrations of energy sources, and mRNA expression levels of related genes, were examined in order to reveal the relationship between boldness and the regulation of energy metabolism in swimming crabs. These results provide a reference for the personality characteristics and underlying mechanisms of crustaceans and a behavioral basis for genetic variation and natural selection [18].

## 2. Materials and Methods

### 2.1. Animal Collection and Maintenance

This experiment was conducted in July and August 2020 at the Key Laboratory of Mariculture, Ministry of Education, Ocean University of China, Qingdao, China. Male swimming crabs (mean carapace width: 100.36 ± 7.23 mm, *N* = 90) were collected from an aquaculture facility in Jiaozhou, Shandong Province. The swimming crabs were housed individually in a glass aquarium (45 × 30 × 30 cm) for two weeks prior to the start of the experiment. During the acclimatization period, one crab was housed in each aquarium in continuously aerated water held at 24 ± 1 °C and 30‰ salinity under a 12 L:12 D photoperiod. Water was changed at a rate of 1/3 per day using seawater that was aerated for 24 h, filtered with an 80-mesh strainer, and adjusted to the target temperature before use. Manila clams (*Ruditapes philippinarum*) (shell length: 31.0–34.0 mm) were provided once daily (0800), and the shells, excrement, and clams were removed three h after feeding. 

### 2.2. Experimental Process and Sample Collection

**Figure 1:**
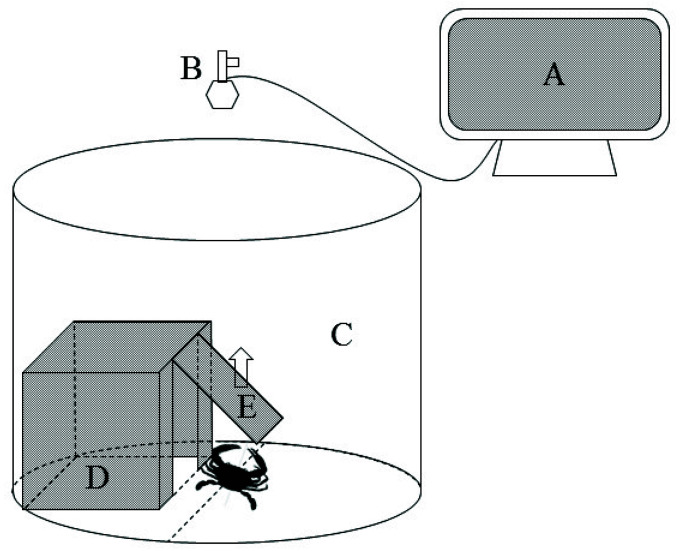
Observation system used to record personality behaviors. Note: (**A**) monitor, (**B**) camera, (**C**) observation box, (**D**) shelter (length × width × height: 30 × 20 × 20 cm), (**E**) trapdoor (length × width: 20 × 20 cm).

After the acclimatization period, healthy swimming crabs (*N* = 43) in the intermolt period with intact appendages were selected for the experiment. The boldness of the swimming crabs was measured using a personality observation system, which consisted of a monitor, camera, trapdoor, shelter, and observation box (Figure 1). The system was not aerated in order to reduce interference during experiments. In order to avoid interference from activities and other distractions, the recordings were carried out in a quiet room under uniform lighting. The lighting and water conditions were the same as during the acclimatization period except that there was no aeration. In this experiment, the boldness of each swimming crab was measured three times within an interval of one week [19]. During the first measurement, fresh seawater was added to the observation box (depth 40 cm), and the swimming crab was placed in a shelter for 10 min for adaptation. After that, the trapdoor was opened, and filming continued for 24 h (Boldness week 1). After the first measurement, the crab was returned to the acclimatization aquarium and housed as before. After one week, the second measurement was conducted using an identical process (Boldness week 2). After another week, the third measurement was conducted. During the third measurement (Boldness week 3), 5 mL of swimming crab hemolymph was added to the seawater in the observation box and mixed gently before recording in order to simulate a dangerous environment [20]. The rest of the process was identical to that of the previous two measurements. A total of 43 individuals were recorded, and their boldness was calculated by quantifying and analyzing the videos. Boldness was calculated as time outside of the shelter/total recording time [21].

After three measurements of boldness, the crabs’ oxygen consumption rates (OCRs) were measured after three days of acclimation. The respiratory container was a beaker (5 L) with connected inlet and outlet pipes sealed with rubber plugs. The water flow rate was adjusted using a valve. Prior to measurement, individuals were housed in the respiratory container for 24 h, and the valve was closed for 45 min for measurement (flowing water method, more details in [22]). In addition, three blank containers were prepared. YSI (Teflo Pro20,YSI Inc., Yellow Springs, USA) was used to measure the concentration of dissolved oxygen in the water at the beginning and end of the measurement period, and the *OCR* was calculated according to the following formula:

$$OCR\left({\mathrm{mgO}}_{2}\cdot g^{-1}\cdot h^{-1}\right)=\left(C_{0}-C_{t}\right)\times V\times W^{-1}\times T^{-1}$$

where *C*_0_ (mg·mL^−1^) represents the oxygen concentration in the blank container, *C_t_* (mg·mL^−1^) represents the oxygen concentration in the respiratory container at the end of the experiment, *V* represents the volume of the respiratory container, *W* represents the wet weight of the crab, and *T* represents the duration of the measurement. 

After *OCR* measurement, crabs were returned to their aquaria and maintained for seven days. After that, crabs were anesthetized in ice and placed on an ice tray, and 500 μL hemolymph was removed from the base of the last pereiopod by piercing the arthrodial membrane with a syringe. The hemolymph was stored in liquid nitrogen. Muscle was removed from both sides of the cheliped, placed in a 1.5 mL centrifugal tube, and stored in liquid nitrogen. After 24 h at 4 °C, the hemolymph was centrifuged at 3000 rpm for 10 min. The supernatant of the hemolymph and muscle was taken and stored at −80 °C until further analysis. The lactate and glucose concentrations in the hemolymph, the glycogen concentration in the muscle, and the relative expressions of mRNA energy-metabolism-related and 5-HT genes in the cheliped muscle were quantified.

### 2.3. Data Collection and Measurement

#### 2.3.1. Determination of Boldness

After the behavior was recorded, three repeated measures of boldness were calculated as time outside of the shelter/total recording time. Only the results of the first measure were used in the bold–shy individual dichotomy. The classification was completed by a personality analysis method based on machine learning (PAML) [4]. In this method, the elbow method was used to find the optimal number of groups. K-means clustering analysis was adopted for the boldness results. After that, support vector machine (SVM), a classical supervised learning algorithm, was used to obtain a maximum-margin hyperplane, separating the samples into different personality (bold/shy) groups based on the distance from the point to the hyperplane [4].

#### 2.3.2. Energy Sources

The glucose and lactate concentrations in hemolymph and the glycogen concentration in the cheliped muscle were measured using commercial assay kits (Nanjing Jiancheng Bioengineering Institute, Nanjing, China). The glucose concentration in hemolymph (mmol·L^−1^) was measured using the glucose oxidase–peroxidase method. The lactate concentration in hemolymph (mmol·L^−1^) was measured using the NBT colorimetric method. The glycogen concentration (mg·g^−1^) in cheliped muscle was measured using the anthrone–sulfuric acid colorimetric method. An automatic microplate reader (Synergy2, Agilent, Santa Clara, CA, USA) was used to read all absorbance values.

#### 2.3.3. mRNA Relative Expression of Energy-Metabolism-Related and 5-HT Genes

- RNA extraction and reverse transcription

Trizol reagent (Vazyme, Nanjing, China) was used to extract total RNA from the cheliped muscle tissue of swimming crabs. The extraction process was carried out on ice. Following extraction, a NanoDrop300 nucleic acid detector was used to detect the concentration and purity of total RNA and ensure that the detection result of A260/A280 = 1.8–2.0; 1% agarose gel electrophoresis was used to evaluate whether the RNA was contaminated or degraded. cDNA was then synthesized using reverse transcription with a HiScript III qPCR Kit (Vazyme, Nanjing, China). After reverse transcription, cDNA was stored at −20 ℃ for subsequent analysis.

- mRNA expression of related genes

mRNA expression of Na^+^/K^+^-ATPase, NADH ubiquinone reductase (NADH-QR), cytochrome c oxidase (CCO), AMPKα, 5-HT, and reference gene β-actin (GenBank accession no. FJ641977) were detected using real-time quantitative RT-PCR (see Table 1 for primer sequence). The CCO (GenBank accession no. ARO92230.1) and NADH-QR (GenBank accession no. 123511446) primer sequences were derived from Ren and Pan [23]. The AMPKα (GenBank accession no. SRP018007) primer sequences were derived from Lu et al. [24]. The Na^+^/K^+^-ATPase primer sequences were derived from Xie et al. [25]. The 5-HT primer sequences were derived from Wang et al. [26].

The fluorescence quantitative determination was carried out using the ChamQ™ SYBR Color reagent (Vazyme, Nanjing, China); 0.4 μL cDNA was used in the measurement. The upstream primer, downstream primer, and corresponding reagent for the corresponding gene were added for amplification. The mRNA relative expression was calculated by 2^−ΔΔCt^. During measurement, the expression specificity of corresponding genes was identified via the melting curve. The amplification system and conditions of RT-qPCR were as follows:

PCR amplification system (20 μL): 2 × ChamQ SYBR Color qPCR Master Mix 10 μL, upstream primer and downstream primer 0.4 μL each, cDNA 0.2 μL, and DEPCH2O 7.2 μL. The PCR amplified condition consisted of pre denaturation (95 °C, 30 s); cyclic reaction (95 °C, 10 s; 60 °C, 30 s), including 30 cycles; melting curve (95 °C, 15 s; 60 °C, 60 s; 95 °C, 15 s); and insulation (4 °C).

### 2.4. Data Analysis

SPSS 24.0 was used for all data analysis. Data are expressed as mean ± standard deviation (mean ± SD). The repeated measurements of boldness were analyzed using the generalized linear mixed model (GLMM). When analyzing boldness, a skewed distribution was used, and the residuals were checked for normal distribution to assess model fit. The ID of crabs was set as the random factor, and the three repeated measure of boldness was set as the fixed factor. Post-hoc comparisons between the three times were also calculated with the calculation module. Other results were analyzed by one-way ANOVA. A Duncan post-hoc comparison test was used to evaluate differences among the three repeated measurements. The correlation between boldness and energy metabolism was analyzed using Spearman’s rank correlation. A chi-squared test was used to compare the numbers of bold and shy individuals. An independent sample *t*-test was used to analyze the differences in energy content and related gene expression between bold and shy individuals. Prior to analysis, the Shapiro–Wilk method was used to test the normality of the data, and Levene’s test was used to examine the homogeneity of variance. Data that did not meet the standards of normality and homogeneity of variance were transformed using arcsine and logarithm; *p* < 0.05 was treated as the significant level of difference in all analyses. 

## 3. Results

### 3.1. Cluster Analysis of Boldness

**Figure 2:**
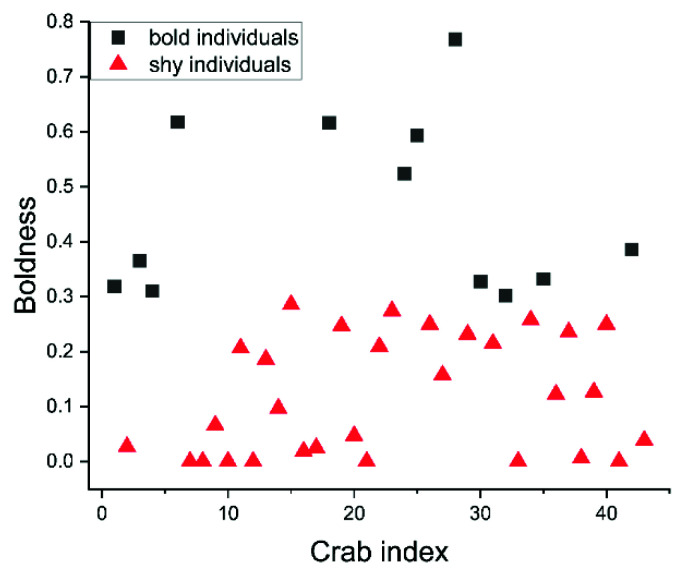
The result of clustering analysis based on boldness.

Crabs were divided into bold and shy individuals based on the K-means clustering results (Figure 2). The boldness scores of bold individuals ranged from about 0.3–1.0, while those of shy individuals mostly ranged from 0.0–0.3 (Figure 2). The number of bold individuals (*N* = 13) was significantly lower than that of shy individuals (*N* = 30) ($\chi_{1,42}^{2}$ = 6.721, *p* < 0.05), and the boldness of bold individuals was significantly higher than that of shy individuals.

### 3.2. Stability of Boldness

**Figure 3:**
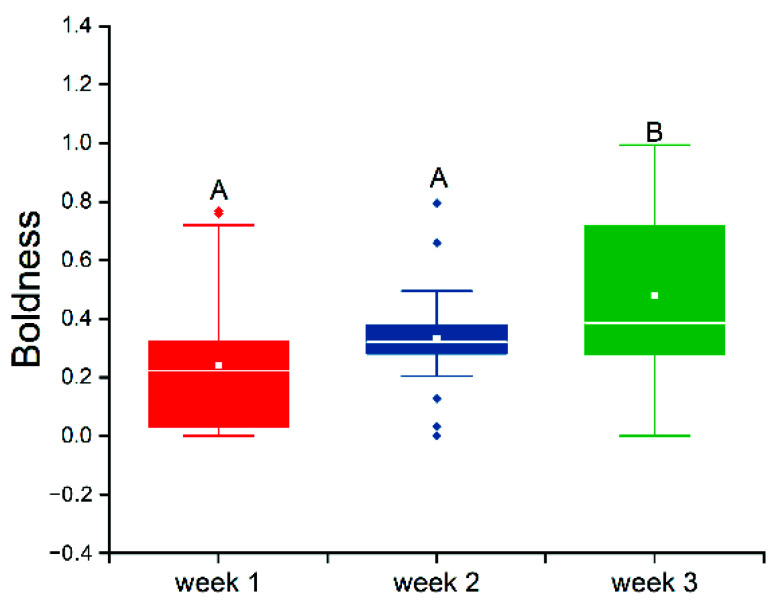
Differences in the results of three repeat boldness tests of swimming crabs. Note: The white line represents the median boldness within the group, and the white rectangle represents the mean boldness within the group. Different capital letters represent significant differences between groups (*p* < 0.05). The diamonds represent outliers in the data.

Repeated measurements showed that boldness increased significantly with time (*F* = 21.502, *p* < 0.001), but there was no significant difference in boldness between the first and the second week (*t* = 4.383, *p* = 0.096). Boldness scores were significantly higher in the third week than in both the first (*t* = 10.504, *p* < 0.001) and the second week (*t* = 6.302, *p* < 0.001) (Figure 3).

### 3.3. OCR

**Figure 4:**
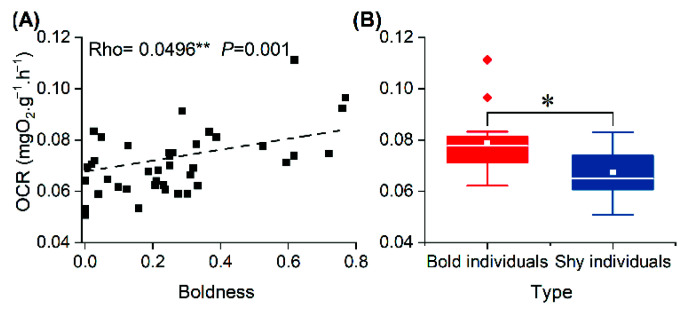
Correlation analysis of OCR and boldness (**A**) and difference in OCR between bold and shy individuals (**B**). Note: The white line represents the median boldness within the group, and the white rectangle represents the mean boldness within the group. Asterisk represents significant differences between groups (“*”: *p* < 0.05; “**”: *p* < 0.01). The diamonds represent outliers in the data.

There was a significant positive correlation between boldness and OCR in swimming crabs. Individuals with high boldness had a higher OCR (Figure 4A), and the OCR of bold individuals was significantly higher than that of shy individuals (*t* = 5.876, *p* = 0.020) (Figure 4B).

### 3.4. Energy Source

**Figure 5:**
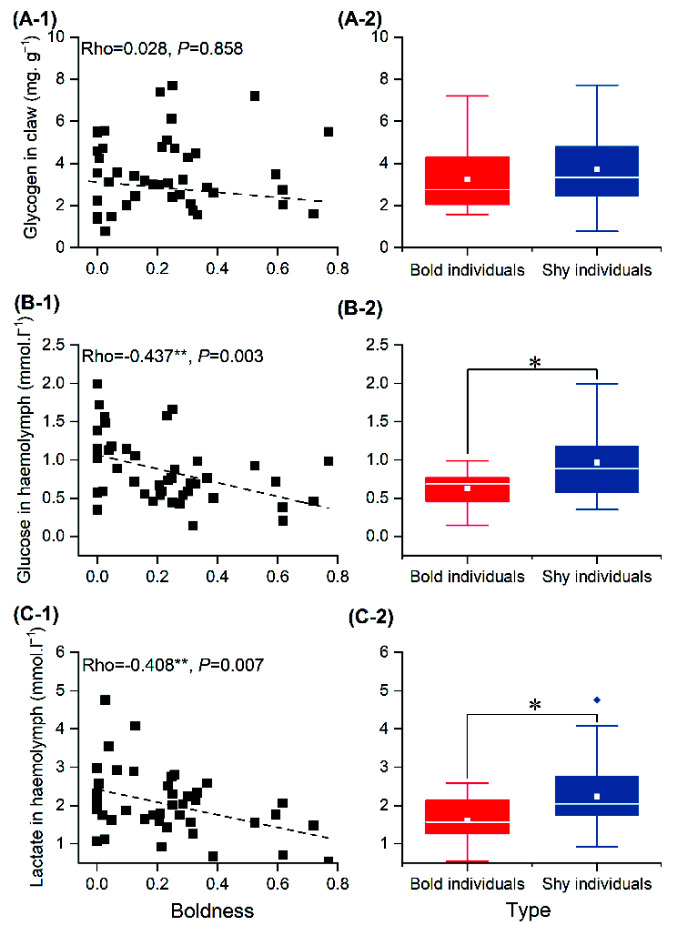
Correlation analysis between boldness and glycogen (**A-1**) in cheliped muscle, glucose (**B-1**) and lactate (**C-1**) in hemolymph, and the corresponding differences between bold and shy individuals (**A-2**,**B-2**,**C-2**). Note: The white line represents the median boldness within the group, and the white rectangle represents the mean boldness within the group. Asterisk represents significant differences between groups (“*”: *p* < 0.05; “**”: *p* < 0.01). The diamonds represent outliers in the data.

There was no significant correlation between boldness and glycogen concentration in cheliped muscle (Figure 5(A-1)). However, there was a significant negative correlation between boldness and glucose and lactate concentrations in hemolymph (Figure 5(B-1,C-1)); individuals with high boldness scores had lower glucose and lactate concentrations in hemolymph. Glucose (*t* = 6.446, *p* = 0.015) and lactate (*t* = 5.452, *p* = 0.025) concentrations in the hemolymph of bold individuals were significantly lower than in the hemolymph of shy individuals (Figure 5(B-2,C-2)), but there was no significant difference in glycogen concentration (*t* = 0.690, *p* = 0.411) (Figure 5(A-2)).

### 3.5. mRNA Relative Expression of Energy-Metabolism-Related and 5-HT Genes

**Figure 6:**
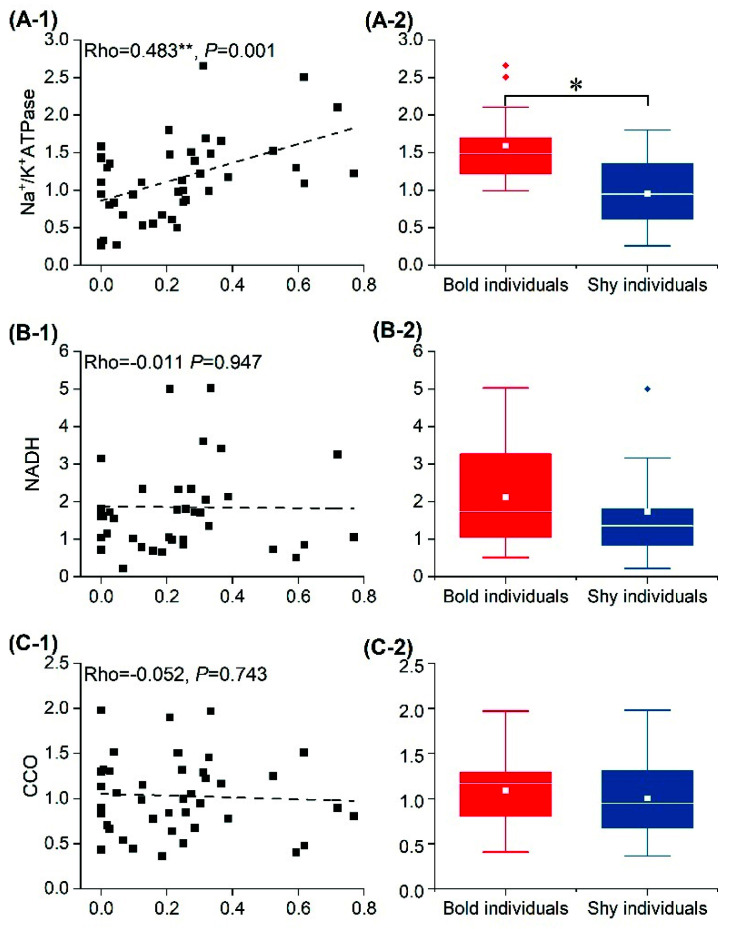

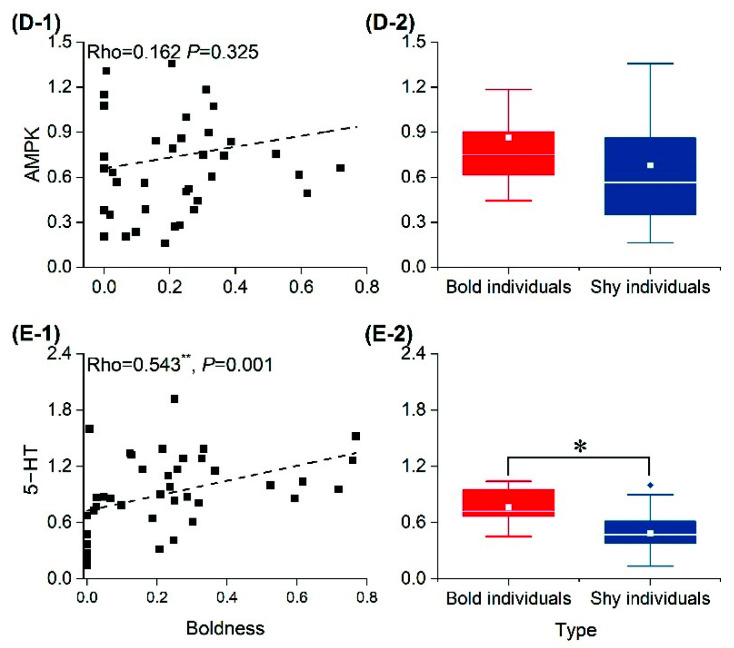
Correlation analysis between boldness and mRNA relative expression levels of Na+/K+-ATPase (**A-1**), NADH (**B-1**), CCO (**C-1**), AMPK (**D-1**), and 5-HT (**E-1**) in cheliped muscle, and differences in mRNA relative expression between bold and shy individuals (**A-2**,**B-2**,**C-2**,**D-2**,**E-2**). Note: The white line represents the median boldness within the group, and the white rectangle represents the mean boldness within the group. Asterisk represents significant differences between groups (“*”: *p* < 0.05; “**”: *p* < 0.01). The diamonds represent outliers in the data.

Boldness was not significantly correlated with the mRNA relative expression of NADH (Figure 6(B-1)), CCO (Figure 6(C-1)), or AMPK (Figure 6(D-1)) in cheliped muscle tissue. The mRNA relative expression of Na^+^/K^+^-ATPase (Figure 6(A-1)) and 5-HT (Figure 6(E-1)) was significantly positively correlated with boldness, and the expression of Na^+^/K^+^-ATPase and 5-HT genes was significantly up-regulated in individuals with high boldness scores. The expression of the two genes in bold individuals was significantly higher than in shy individuals (Na^+^/K^+^-ATPase: *t* = 6.211, *p* = 0.033; 5-HT: *t* = 3.463, *p* = 0.017) (Figure 6(A-2,E-2)), while NADH (*t* = 4.728, *p* = 0.258) (Figure 6(B-2)), CCO (*t* = 2.571; *p* = 0.772) (Figure 6(C-2)), and AMPK (*t* = 2.082, *p* = 0.344) (Figure 6(D-2)) did not differ significantly among groups.

## 4. Discussion

The balance between personality stability and behavioral plasticity has become a growing area of interest for researchers [27]. In a relative study of crustaceans, Hazlett first observed both personality stability and behavioral plasticity in hermit crabs (*Pagurus bernhardus*) [20]. Recent studies have confirmed that the boldness of hermit crabs (*P. bernhardus*) does not change when the color of their shells changes [28]. However, unsuitable temperatures can change intraindividual variation in exploration behavior between individuals [29]. In this study, differences in boldness between individual swimming crabs were significant (Figure 2) and stable across the first two repeated measurements. During the third measurement, this time in a seemingly dangerous situation, boldness scores significantly increased (Figure 3). This showed both personality stability and behavioral plasticity in swimming crabs. In this study, the boldness of bold and shy crabs increased significantly in cases of danger; the possible reason is that, as a species with high aggression, frequent movement and higher boldness may be an effective means to respond to danger [30,31]. This result also shows that a different personality does not affect plasticity when at risk.

Many studies have shown that the formation and maintenance mechanisms of personality are closely related to energy metabolism [15,32]. This study found that boldness was significantly positively correlated with OCR, and the OCR of bold individuals was significantly higher than that of shy individuals (Figure 4), indicating that metabolic rate differences may be closely related to differences in boldness [33,34]. There was no significant relationship between boldness and glycogen content in cheliped muscle, and there was no significant difference in glycogen content between bold and shy individuals. This may be because swimming crabs in this experiment were provided with adequate clams, leading to almost identical concentrations of the major energy storage molecules (glycogen) in the bold and shy individuals [35]. This finding is similar to that in blue crabs (*Panopeus herbstii*) [21]. The boldness of swimming crabs was significantly negatively correlated with the concentrations of both glucose and lactate in hemolymph (Figure 5(B-1,C-1)), such that the concentrations of glucose and lactate in the hemolymph of bold crabs were significantly lower than those of shy crabs (Figure 5(B-2,C-2)). This is consistent with the result of OCRs of bold crabs being higher than those of shy crabs (Figure 4). This may be because the metabolic rates of bold individuals are higher than those of shy individuals, resulting in the difference in glucose concentrations in hemolymph [36]; this is consistent with research investigating OCRs and personality in carp (*Cyprinus carpio*) [37]. Lactate is produced through anaerobic respiration, and lactate content reflects the energy supply intensity of anaerobic respiration. In this study, the boldness of swimming crabs was significantly positively correlated with lactate concentrations in hemolymph, indicating higher anaerobic respiration intensity in bold individuals. These results are consistent with our previous findings that anaerobic respiration intensity in bold swimming crabs is higher while fighting [38]. Therefore, the differences in metabolic rate and energy substance content may be one of the internal mechanisms driving the formation and maintenance of boldness in swimming crabs.

The energy state of an individual is the result of energy production and consumption [39]; 90% of ATP is produced by the oxidative phosphorylation pathway of mitochondria [26]. CCO and NADH, two key enzymes regulating this process, are closely related to individuals’ levels of oxidative phosphorylation [40]. The results of this experiment showed that there was no correlation between the boldness of crabs and the relative expression of CCO and NADH mRNA in muscle tissue (Figure 6(B-1,C-1)), and there was no significant difference in mRNA relative expression between bold and shy individuals (Figure 6(B-2,C-2)), indicating that energy production is not related to differences in boldness. AMPK is a key protein in the regulation of individual energy metabolism [41]. In this study, there was no significant correlation between the mRNA relative expression of AMPK and boldness (Figure 6(D-1)), nor was there a significant difference in the mRNA relative expression of AMPK between bold and shy individuals (Figure 6(D-2)), indicating that ATP production in the muscle tissue did not cause the difference in boldness. Na^+^/K^+^-ATPase is one of the important proteins involved in ion transportation, representing individual energy consumption [42]. In this study, the mRNA relative expression of Na^+^/K^+^-ATPase was significantly correlated with boldness (Figure 6(A-1)) and the mRNA relative expression in the bold individuals was significantly higher than in the shy individuals (Figure 6(A-2)), indicating that the bold individuals consumed more energy than the shy individuals. Thus, the difference in the regulation of energy metabolism between bold and shy individuals lies in energy consumption rather than energy production.

Personality differences are influenced not only by energy metabolism but also by neurotransmitters and hormones [43,44]. Studies have shown that increased 5-HT content can significantly reduce aggression in vertebrates [45] but significantly increase the heart rate, activity, and sociality of crayfish (*Procambarus clarkii*) [16]. In this study, the mRNA relative expression of 5-HT genes was significantly correlated with boldness (Figure 6(E-1)), and the mRNA relative expression of bold individuals was significantly higher than that of shy individuals (Figure 6(E-2)), indicating that 5-HT may significantly increase the boldness of swimming crabs. The differences in mRNA relative expression may be another cause of personality difference.

## 5. Conclusions

This study has demonstrated that boldness in *P. trituberculatus* exhibits personality stability across repeated measurements and behavioral plasticity in a dangerous situation. Individual boldness is significantly correlated with OCR, glucose concentration, and lactate concentration, and the difference in energy metabolism regulation lies in energy consumption, which may be the internal mechanism underlying differences in the boldness of swimming crabs. In addition, the relative expression of 5-HT gene mRNA may be another mechanism driving these differences. The formation and maintenance of animal personality, leading to stable behavioral and physiological differences (which is an influence factor generating variation [18]), is an important factor affecting evolution. The factors influencing animal personality are complex [15,46]. Much more research is needed to fully elucidate the mechanisms of animal personality formation, maintenance, and change. 

## Figures and Tables

**Table 1:** Sequences of specific primers.

Genes	Primer Sequence	
*CCO*	Forward	5′-GGCTACCAGCAGTGACA-3′
	Reverse	5′-ATGGCTGCCTCTCTCACCT-3′
*NADH-QR*	Forward	5′-CAATCATCGCACAGGAGAG-3′
	Reverse	5′-CCAGACGCTGTCGTTTCAA-3′
*Na^+^/K^+^-ATPase*	Forward	5′-TACAAGAACGGAGACAACGA-3′
	Reverse	5′-TCAAGCGACACACTTTCCTCT-3′
*AMPKα*	Forward	5′-5GCAATAGCAGGCGTACAACA-3′
	Reverse	5′-CTTCTGCCCTTTGATGATGAG-3′
*5-Hydroxytryptamine receptor 1*	Forward	5’-TGTTAGCATTGCCCAGGT-3′
	Reverse	5′-AGTCTCTATCCCGAGGTTCTAC-3′
*β-actin*	Forward	5′-TGCTGTCCTTGTACGCCTCC-3′
	Reverse	5′-CAGACGCAGGATAGCGTGA-3′

## Data Availability

The data presented in this study are available in the article. Further information is available upon request from the corresponding author.
